# Ethnic inequalities in coverage and use of women’s cancer screening in Peru

**DOI:** 10.1186/s12905-024-03225-6

**Published:** 2024-07-24

**Authors:** Claudio Intimayta-Escalante

**Affiliations:** 1 Facultad de Medicina de San Fernando, Universidad Nacional Mayor de San Marcos, Lima, Peru; 2 Departamento de Promoción de la Salud, Prevención y Control Nacional del Cáncer, Instituto Nacional de Enfermedades Neoplásicas, Lima, Peru

**Keywords:** Early detection of cancer, Sociodemographic factors, Health inequities, Ethnicity, Peru

## Abstract

**Objective:**

This study aimed to assess ethnic inequalities in the coverage and utilization of cancer screening services among women in Peru.

**Methods:**

Data from the 2017–2023 Demographic and Family Health Survey in Peru were analyzed to evaluate ethnic disparities in screening coverage for breast and cervical cancer, including clinical breast examination (CBE), Pap smear test (PST), and mammography. Measures such as the GINI coefficient and Slope Index of Inequality (SII) were used to quantify coverage and utilization disparities among ethnic groups.

**Results:**

The study included 70,454 women aged 30–69. Among women aged 40–69, 48.31% underwent CBE, 84.06% received PST, and 41.69% underwent mammography. It was found inequalities in coverage for any cancer screening (GINI: 0.10), mammography (GINI: 0.21), CBE (GINI: 0.19), and PST (GINI: 0.06), in 25 Peruvian regions. These inequalities were more pronounced in regions with larger populations of Quechua, Aymara, and Afro-Peruvian women. In rural areas, Quechua or Aymara women (SII: -0.83, -0.95, and − 0.69, respectively) and Afro-Peruvian women (SII: -0.80, -0.92, and − 0.58, respectively) experienced heightened inequalities in the uptake of CBE, mammography, and PST, respectively. Like Quechua or Aymara women (SII: -0.50, SII: -0.52, and SII: -0.50, respectively) and Afro-Peruvian women (SII: -0.50, SII: -0.58, and SII: -0.44, respectively) with only a primary education.

**Conclusion:**

Ethnic inequalities affect breast and cervical cancer screening coverage across regions in Peru. In Quechua, Aymara, and Afro-Peruvian women the uptake of mammography, CBE, and PST was less frequently than their white or mestizo counterparts. These inequalities are attributed to sociodemographic conditions such as lower education levels and residence in rural or non-capital areas.

**Supplementary Information:**

The online version contains supplementary material available at 10.1186/s12905-024-03225-6.

## Background

In 2022, breast cancer impacted over two million women globally, resulting in an age-standardized mortality rate of 12.7 deaths per 100,000 women [1]. Similarly, cervical cancer affected 600,000 women, with an age-standardized mortality rate of 7.1 deaths per 100,000 women [2]. However, Peru presents a hopeful trend, with 68% of women aged 25 to 65 having undergone cervical cancer screening, a vital practice for early detection and treatment [3]. Notably, although 84% of Peruvian women undergo this screening, the mortality rate for cervical cancer remains high at 12.1% [2, 4]. Furthermore, despite breast cancer ranking as the second most diagnosed neoplasm in Latin America [2, 5], only 16.9% of Peruvian women aged 40–59 had received a mammogram by 2018, and by 2022, the mortality rate for breast cancer stood at 9.4 [2, 6].

Guidelines advocate for regular screenings for women aged 40 to 74, with mammograms recommended every one to two years [7]. Women aged 30–49 should undergo at least one cervical cancer screening test [8], such as the Papanicolaou test (commonly known as the pap smear test). Detecting cancer early through this proactive approach significantly improves patient survival rates. However, screening programs in low- and middle-income countries encounter challenges in reaching the entire target population [9–11]. Peru faces obstacles as approximately 50% of breast cancer cases are identified at an advanced stage [12]. Responding to these challenges, Peru has revised its breast and cervical cancer screening programs since 2017 to address identified gaps [13]. The age ranges for clinical breast examinations have been adjusted to 40 to 69 years, for pap smears 50 to 64 years, and for mammograms 50 to 69 years, with the aim of achieving 60% coverage within the target age group [14, 15].

Despite significant investment, disparities in cervical and breast cancer screening services persist in Peru [16, 17]. Contributing factors include inadequate resources, infrastructure limitations, sociodemographic conditions (such as age, income, education, and area of residence), as well as health and personal factors like awareness, language barriers, and cultural beliefs [18–21]. These disparities disproportionately impact women with lower socioeconomic status, leading to increased mortality rates from breast and cervical cancer [22, 23]. While investigations in Latin America typically focus on sociodemographic factors, ethnicity is often overlooked in studies [24, 25]. In Peru, a multicultural nation where ethnic identification significantly impacts healthcare access [26, 27], it is crucial to examine ethnic disparities in cancer care. Therefore, this study aims to assess ethnic inequities in the coverage and utilization of cancer screening services among women in Peru.

## Methods

### Study design

A cross-sectional study was conducted using data from the Demographic and Family Health Survey (DHS, or ENDES in Spanish) spanning from 2017 to 2023, with a focus on cancer screening coverage among women across 25 regions in Peru. The National Institute of Statistics and Informatics (or INEI acronym in Spanish) annually conducts the DHS survey nationwide. Peru, a populous country in Latin America with approximately 32 million inhabitants, harbors a significant female population, with a notable concentration, particularly among women, in the capital city of Lima [28]. This research encompassed an analysis of general cancer screenings, clinical breast examinations, Papanicolaou smear tests, and mammography performance [14, 15].

### Cancer screening evaluations

The DHS survey includes specific inquiries designed to gather data concerning cancer screening among Peruvian women. These inquiries cover general cancer screening for women aged 30 to 69 (Have you ever undergone a general cancer screening?), clinical breast examinations for women aged 40 to 69 (Have you had a clinical breast exam performed by a physician or other healthcare professional?), Papanicolaou smear tests for women aged 50 to 64 (Have you had a Pap smear test performed by a physician or other healthcare professional?), and mammography for women aged 50 to 69 within the last 24 months (Have you had a mammogram performed by a physician or other healthcare professional?).

### Sociodemographic conditions

It was explored how various sociodemographic factors influenced cancer screening coverage or evaluations among women. These characteristics included educational level (none, elementary school, high school, or university), wealth index (ranging from the lower to the first quintile), residency area (urban or rural) and place (capital or other regions), health insurance (with or without), and ethnic groups in Peru (white, mestizo, Quechua or Aymara, and Afro-Peruvian).

### Statistical analysis

The analysis was performed using Stata v.17.0, which considered the complex sample of DHS by adjusting for survey design features with the “svy” command. Descriptive statistics, including frequencies and percentages, were calculated for each categorical variable. The Rao-Scott test was utilized to detect notable variations among women who received cancer screening evaluations.

### Inequality analysis in coverage

The “lorenz” command was employed to assess distributional inequalities. It was examined ethnic and sociodemographic inequalities in cancer screening coverage among women in 25 Peruvian regions. The GINI coefficient is determined using a graph with two axes and an equidistant line, known as the “equity line,” with a GINI value of zero. To calculate the GINI coefficient, it is necessary to divide the area between the equity line and the Lorenz curve (which illustrates the distribution of cancer coverage in regions) by the total area under the equity line. A GINI coefficient of zero indicates perfect equity, whereas a value of one signifies absolute inequality [29].

### Inequality analysis in evaluations

It was also used the “siilogit” command to look at differences between individuals using the Slope Index of Inequality (SII) based on ethnic group and a stratified analysis by sociodemographic conditions to assess differences in how well Peruvian women did on cancer screening evaluations. The SII enables us to include a wealth index as an equity stratifier and calculate inequality with values ranging from − 1 to + 1, where values between − 1 and 0 indicate higher disparity and values between 0 and + 1 represent lower inequality [30].

### Sensitivity analysis

Additionally, it was created plots in R Studio v.4.2.2 to represent the geographic distribution of cancer screening coverage among women in 25 regions of Peru. Also, to assess the impact of the COVID-19 pandemic, it was created an annual variation plot showing changes in coverage, GINI index, and SII for cancer screening assessments from 2017 to 2023.

### Ethical aspects

Given that the DHS data collection process involved participants’ informed consent, no ethical committee evaluation was necessary. Moreover, data obtained from the INEI platform are anonymized and securely stored (http://iinei.inei.gov.pe/microdatos/).

## Results

The study included 70,454 Peruvian women aged 30 to 69 (Appendix 1). Among them, 34.34% (95% CI: 33.69 to 35.00) had undergone cancer screening. Specifically, 48.31% (95% CI: 47.71 to 49.21) of women aged 40 to 69 had received clinical breast examinations by healthcare professionals. Similarly, 84.06% (95% CI: 83.22 to 84.86) of women aged 50 to 64 had undergone Pap smear tests administered by healthcare providers. Additionally, 41.69% (95% CI: 40.51 to 42.89) of women aged 50 to 69 had received mammograms from healthcare professionals within the preceding 24 months.

Among the women aged 30 to 69, the majority had attained at least a high school education (67.31%) and belonged to the first three wealth quintiles (63.23%). Additionally, a significant proportion resided in urban areas (81.25%) outside the capital (59.69%) and had health insurance coverage (83.24%). Ethnic identification revealed 47.25% mestizo, 25.27% Quechua, 10.33% Afro-Peruvian, 7.22% white, and 2.13% Aymara (Table 1).

**Table 1:** Sociodemographic characteristics of Peruvian women with cancer screening evaluations according to ethnic identifications

Variables	Total of Peruvian women aged 30–69 (*n* = 70,454)		Women aged 30–69 screened for cancer (*n* = 19,839)		Women aged 40–69 with clinical breast exam (*n* = 13,852)		Women aged 50–64 with PAP smear test (*n* = 12,211)			Women aged 50–69 with mammography (*n* = 4,741)		
	*n*	%*(95%IC)	*n*	%*(95%IC)	*n*	%*(95%IC)	*n*	%*(95%IC)	*n*		%*(95%IC)	
**Educational level**												
Without	4315	5.01 (4.76 to 5.27)	638	17.2 (15.39 to 19.18)	493	18.96 (16.79 to 21.34)	1104	62.46 (59.22 to 65.59)	202		12.5 (10.33 to 15.04)	
Elementary	21,211	25.71 (25.14 to 26.28)	4624	24.72 (23.77 to 25.71)	3584	30.71 (29.45 to 32.01)	4851	76.6 (75.00 to 78.13)	1402		22.97 (21.44 to 24.57)	
High School	24,901	36.59 (35.93 to 37.26)	7194	34.03 (32.99 to 35.08)	4868	51.69 (50.15 to 53.23)	3628	89.55 (88.20 to 90.77)	1933		46.8 (44.61 to 49.01)	
University	20,027	32.69 (31.98 to 33.41)	7383	44.9 (43.63 to 46.17)	4907	69.56 (68.04 to 71.05)	2628	93.09 (91.66 to 94.30)	2200		71.86 (69.54 to 74.06)	
**Wealth Index**												
Q1 (Poortest)	21,161	17.67 (17.14 to 18.22)	4488	22.6 (21.71 to 23.52)	2194	18.72 (17.72 to 19.76)	3415	65.8 (63.98 to 67.58)	554		8.41 (7.51 to 9.41)	
Q2	17,053	19.09 (18.56 to 19.64)	4576	29.72 (28.62 to 30.85)	2694	36.99 (35.35 to 38.66)	2535	78.43 (76.32 to 80.40)	928		25.47 (23.39 to 27.66)	
Q3	13,438	20.59 (20.06 to 21.13)	3955	32.57 (31.28 to 33.88)	2880	46.23 (44.35 to 48.13)	2264	85.41 (83.34 to 87.27)	1151		37.52 (35.01 to 40.09)	
Q4	10,693	21.01 (20.41 to 21.63)	3590	37.31 (35.81 to 38.84)	2998	57.24 (55.25 to 59.21)	2181	91.06 (89.39 to 92.48)	1446		51.59 (48.90 to 54.27)	
Q5 (Richest)	8109	21.63 (20.88 to 22.4)	3230	46.78 (45.02 to 48.55)	3086	73.96 (72.06 to 75.77)	1816	94.12 (92.39 to 95.48)	1658		72.32 (69.67 to 74.82)	
**Residency Area**												
Rural	23,522	18.75 (18.15 to 19.36)	5469	25.27 (24.37 to 26.18)	2959	22.37 (21.31 to 23.47)	4026	69.04 (67.37 to 70.66)	904		12.35 (11.30 to 13.47)	
Urban	46,932	81.25 (80.64 to 81.85)	14,370	36.42 (35.65 to 37.20)	10,893	54.41 (53.37 to 55.44)	8185	87.61 (86.66 to 88.50)	4833		48.76 (47.37 to 50.16)	
**Residency Place**												
Others regions	59,918	59.69 (58.63 to 60.74)	16,451	31.56 (30.97 to 32.16)	10,774	39.06 (38.25 to 39.89)	10,059	78.32 (77.36 to 79.25)	4205		30.58 (29.55 to 31.64)	
Capital	10,536	40.31 (39.26 to 41.37)	3388	38.49 (37.14 to 39.86)	3078	61.98 (60.21 to 63.72)	2152	92.38 (90.84 to 93.68)	1532		57.8 (55.44 to 60.12)	
**Ethnic Group?**												
White or Mestizo	27,133	57.75 (57.02 to 58.48)	9298	37.35 (36.39 to 38.31)	6899	57.38 (26.09 to 58.66)	5708	88.46 (87.38 to 89.47)	3416		50.88 (49.20 to 52.56)	
Quechua or Aymara	21,632	29.05 (28.37 to 29.75)	6186	30.62 (29.48 to 31.78)	3536	37.43 (35.87 to 39.01)	4382	79.74 (78.17 to 81.22)	1474		31.20 (29.20 to 33.27)	
Afro-Peruvian	5687	10.95 (10.53 to 11.39)	1554	27.94 (26.21 to 29.75)	1077	38.48 (36.04 to 40.98)	1162	79.50 (76.62 to 82.10)	464		30.85 (27.76 to 34.11)	
Others	1708	2.24 (2.04 to 2.46)	357	24.89 (21.40 to 28.74)	225	42.33 (36.02 to 48.91)	251	74.55 (67.51 to 80.51)	101		37.59 (28.69 to 47.42)	
**Health Insurance?**												
Don´t Have	8445	16.76 (16.22 to 17.31)	1829	24.8 (23.26 to 26.40)	1636	43 (40.74 to 45.29)	1798	76.17 (73.71 to 78.48)	657		30.81 (28.10 to 33.67)	
Have	50,896	83.24 (82.69 to 83.78)	16,365	35.45 (34.72 to 36.20)	10,735	49.82 (48.78 to 50.85)	10,413	85.84 (84.99 to 86.65)	5080		44 (42.72 to 45.28)	

*Weighted estimation for complex sample

*Note* In the comparison between women according to the performance of cancer screening evaluation with Rao-Scott test, all *p*-values were less than 0.001

Regarding cancer screening evaluations in target age groups based on sociodemographic variables. The study indicated substantial variations in the percentage of women who received examinations compared to those who did not (*p* < 0.001). Following wealth quintile and residence, education was the variable with the greatest variation in cancer screening evaluation performance (Table 1).

**Fig. 1:**
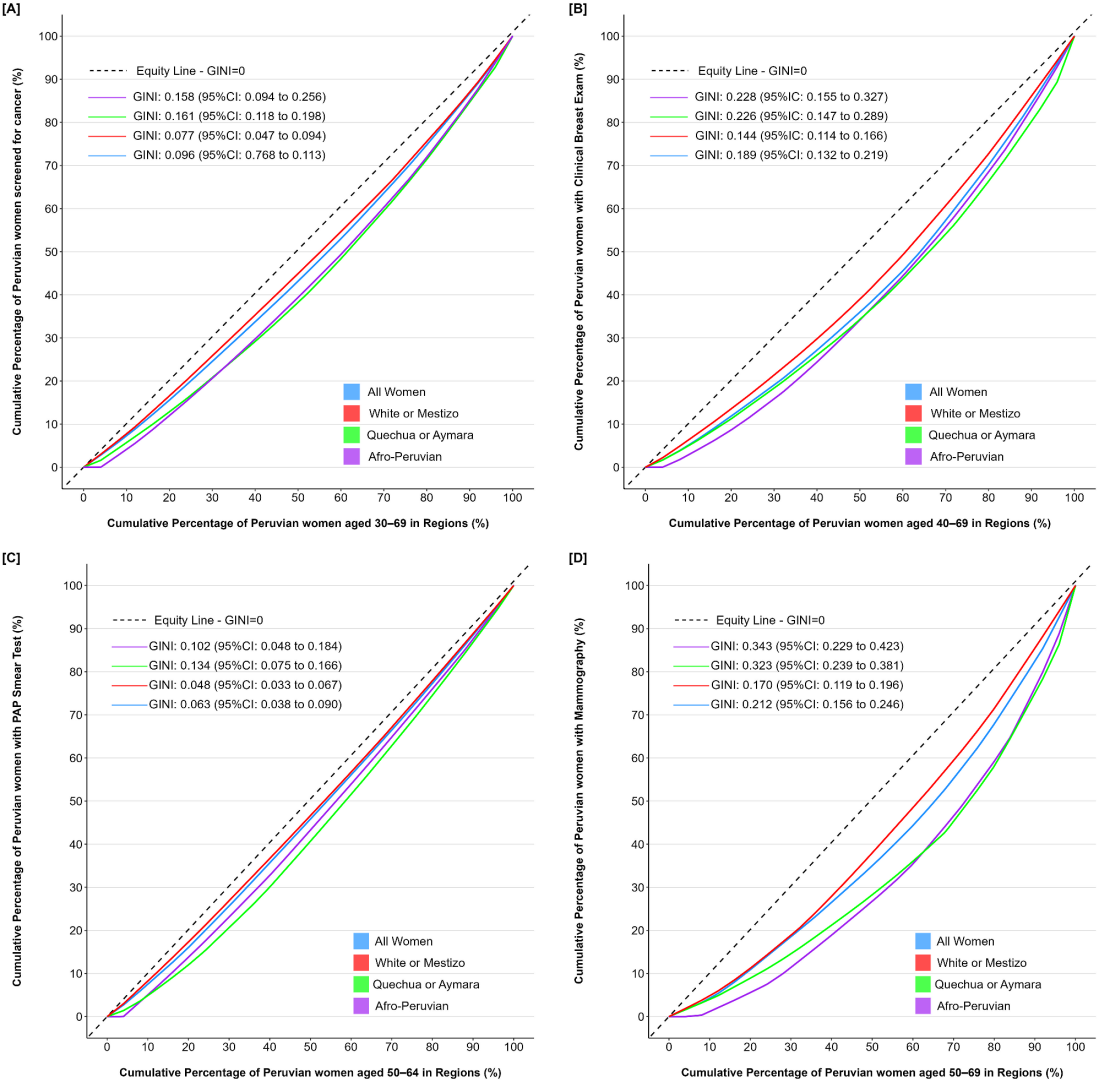
Inequality in women’s cancer screening coverage among different ethnic groups in the 25 Peruvians regions

The study delved into inequality in cancer screening coverage across target age groups of Peruvians women evaluated in 25 regions (Appendix 2). Screening coverage for any cancer among women aged 30–69 revealed a GINI coefficient of 0.10 (95% CI: 0.08 to 0.11). For women aged 50 to 64, Pap test coverage exhibited a GINI coefficient of 0.06 (95% CI: 0.04 to 0.09), while clinical breast examination coverage for women aged 40 to 69 demonstrated a GINI coefficient of 0.19 (95% CI: 0.13 to 0.22). Moreover, mammography coverage among women aged 50 to 69 displayed a GINI coefficient of 0.21 (95% CI: 0.16 to 0.25), with higher inequality observed in regions with larger populations of Quechua, Aymara, and Afro-Peruvian women. But the inequality in coverage for different ethnic groups of women across the 25 regions is higher (Fig. 1).

**Fig. 2:**
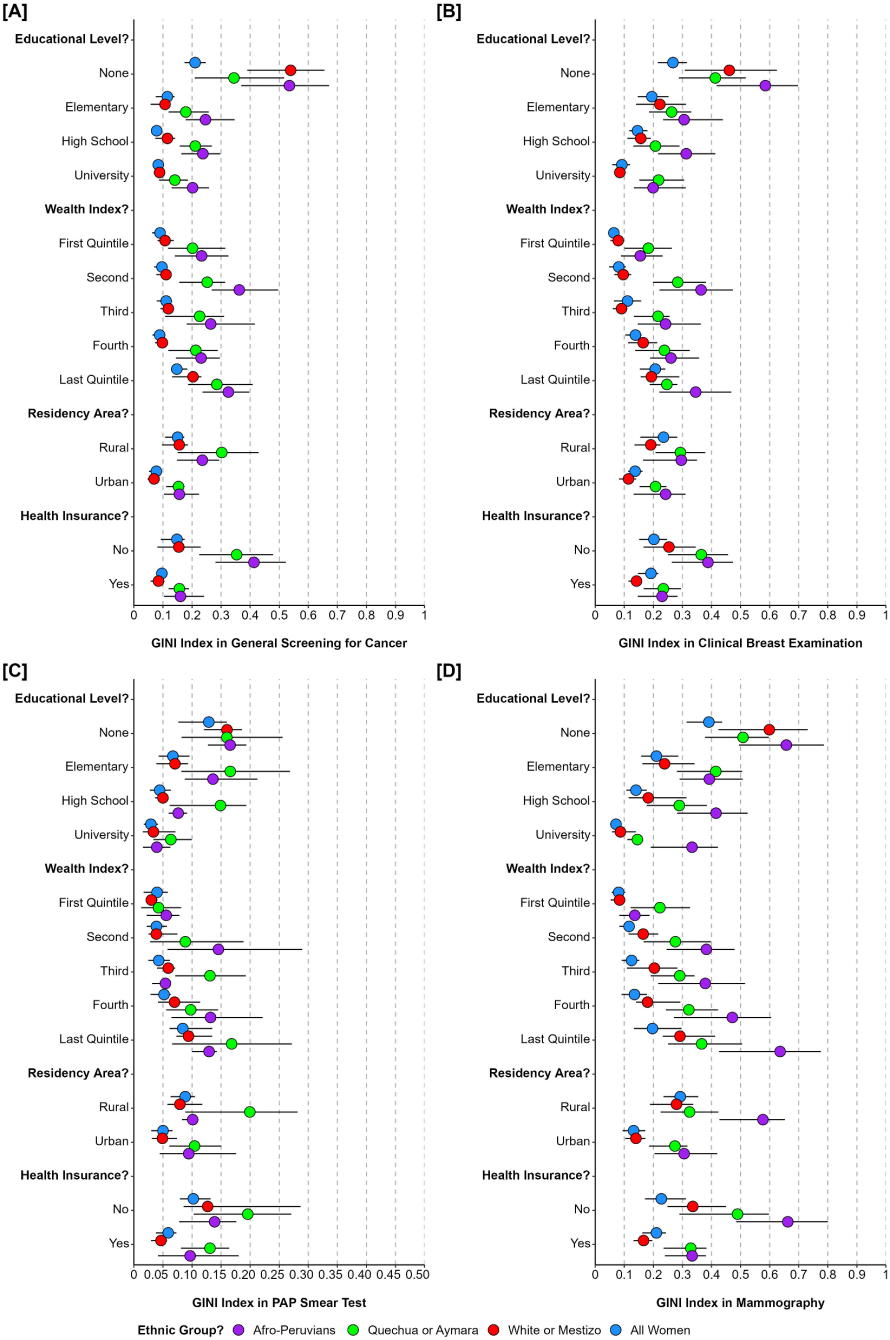
Inequality in women’s cancer screening coverage among the 25 Peruvians regions according to sociodemographic conditions

In the analysis of ethnic inequalities in cancer screening coverage among women of target ages groups in 25 regions (Fig. 2). Greater inequality in cancer screening coverage among women aged 30 to 69 in regions with white or mestizo women (GINI: 0.54, 95%CI: 0.39 to 0.66) and Afro to Peruvian women (GINI: 0.53, 95%CI: 0.37 to 0.67) without education. The inequality in clinical breast examination coverage among all women aged 40 to 69 was greater in regions with Afro-Peruvians (GINI: 0.59, 95%CI: 0.42 to 0.70) and white or mestizo women (GINI: 0.46, 95%CI: 0.31 to 0.63) without education. Similarly, inequality in Pap smear coverage among women aged 50 to 64 was higher in regions with Quechua or Aymara women in rural areas (GINI: 0.20, 95%CI: 0.09 to 0.28) and without health insurance (GINI: 0.20, 95%CI: 0.10 to 0.27). Inequality in mammography coverage was also identified in women aged 50 to 69, with greater inequality in regions with Afro-Peruvians women without health insurance (GINI: 0.66, 95%CI: 0.49 to 0.80), in the last wealth quintile (GINI: 0.64, 95%CI: 0.43 to 0.78), and without education (GINI: 0.66, 95%CI: 0.49 to 0.79).

**Fig. 3:**
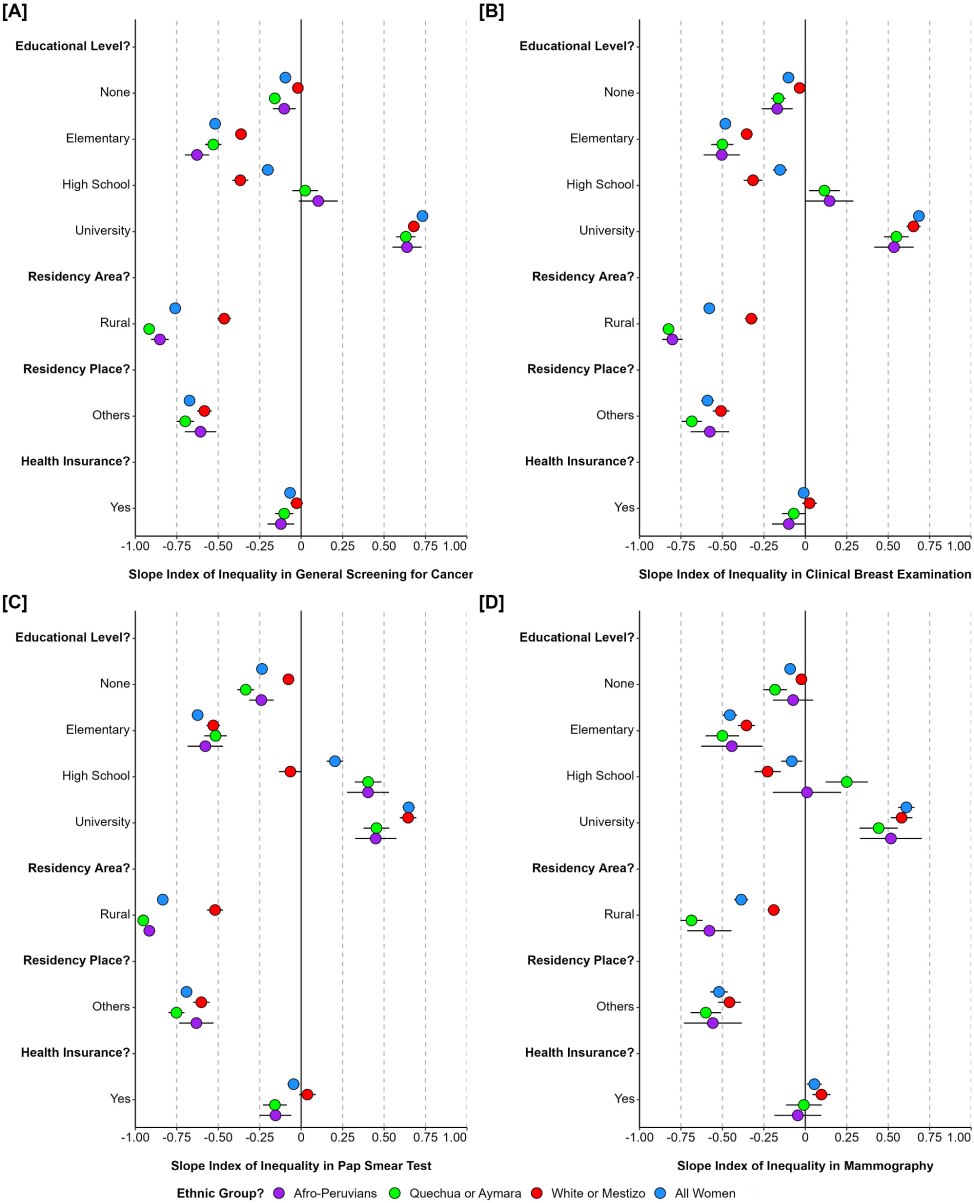
Inequality in cancer screening services use among Peruvians women according to sociodemographic conditions

Examining sociodemographic conditions related to ethnic inequalities (Fig. 3). Quechua or Aymara, and Afro-Peruvian women in rural areas had more inequality to uptake general cancer screening (SII: -0.92, and SII: -0.85, respectively), clinical breast examination (SII: -0.83, and SII: -0.80, respectively), and pap smear test (SII: -0.95, and SII: -0.92, respectively). Also, Quechua or Aymara women living outside capital had more inequality to get uptake general cancer screening (SII: -0.70, 95%CI: -0.75 to -0.65), clinical breast examination (SII: -0.69, 95%CI: -0.75 to -0.62), pap smear test (SII: -0.75, 95%CI: -0.80 to -0.70), and mammography (SII: -0.60, 95%CI: -0.69 to -0.51). As well as for Quechua or Aymara, and Afro-Peruvian women with only elementary education had more inequality to get general cancer screening (SII: -0.53, and SII: -0.63, respectively), clinical breast examination (SII: -0.50, and SII: -0.50, respectively), pap smear test (SII: -0.52, and SII: -0.58, respectively), and mammography (SII: -0.50, and SII: -0.44, respectively). Health insurance affiliation, half inconsistent scenarios in ethnic inequalities in the coverage and use of cancer screening services.

**Fig. 4:**
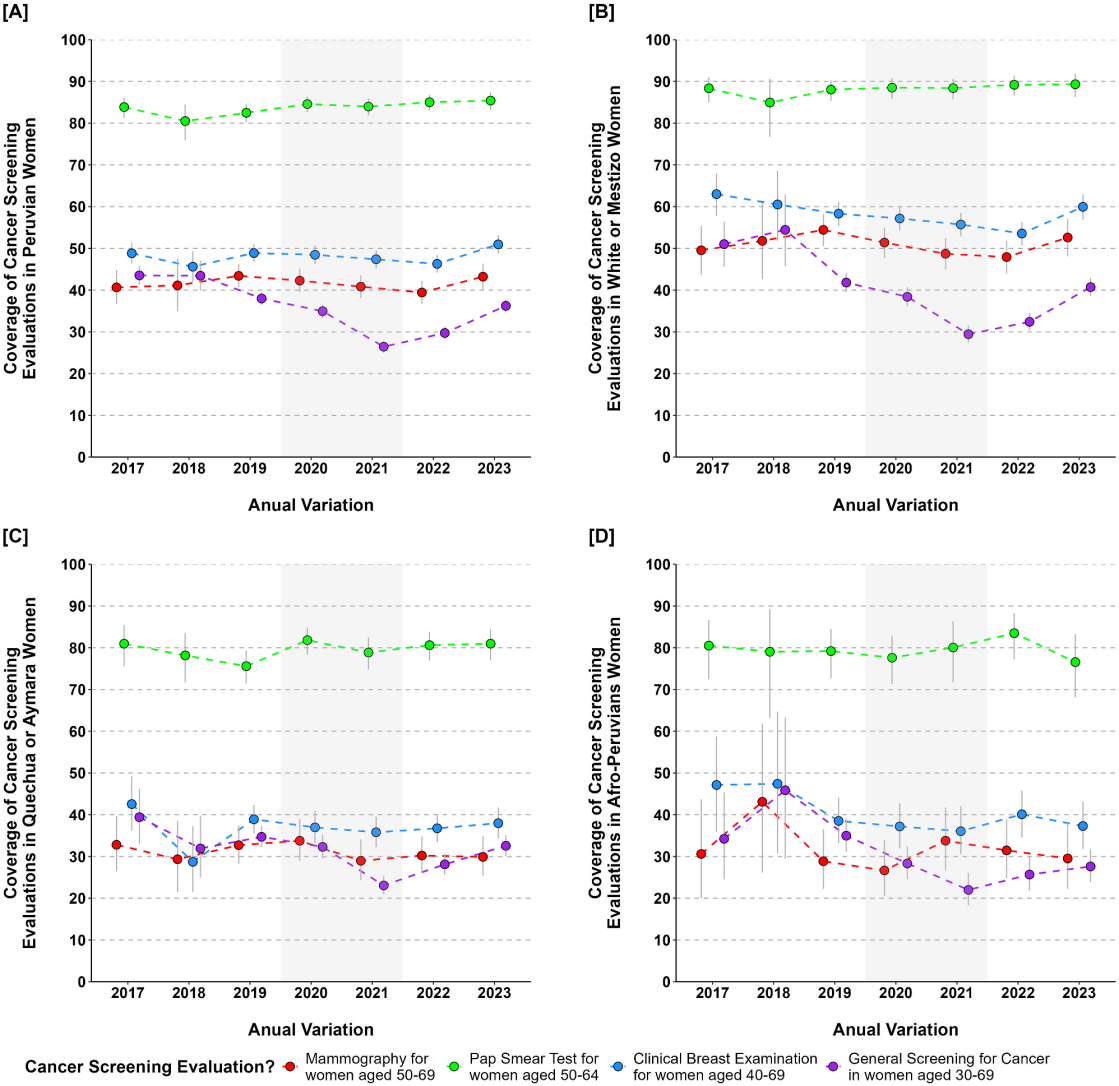
Annual variations in cancer screening coverage for women among ethnic groups. *Note* Peru was affected by the COVID-19 pandemic over the period represented in gray

**Fig. 5:**
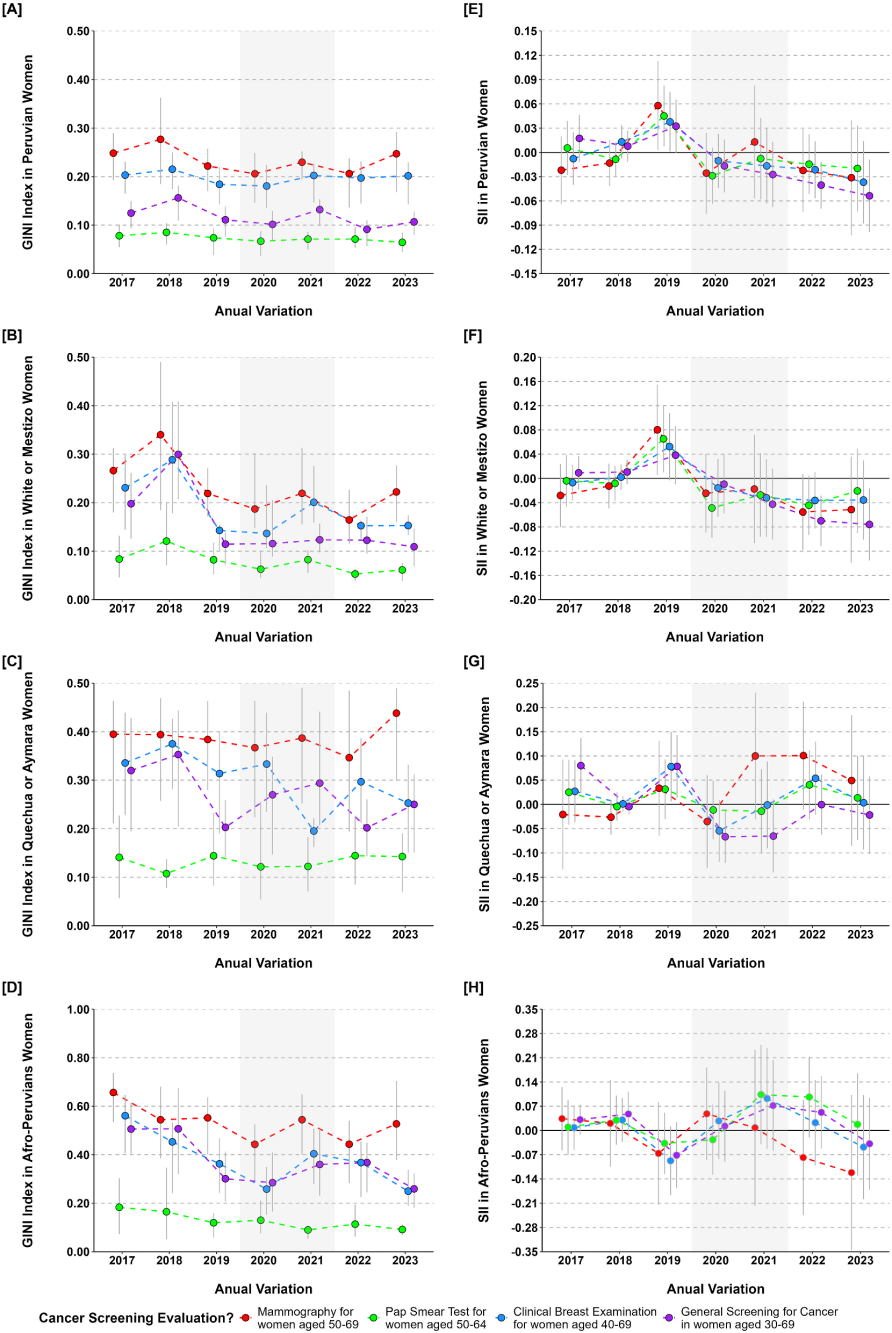
Annual variations of inequality in cancer screening for women among ethnic groups. *Note* Peru was affected by the COVID-19 pandemic over the period represented in gray

In the analysis of annual variations in cancer screening coverage for certain age groups of women in the 25 regions (Fig. 4). Pap smear coverage was almost uniform, at approximately 70%. Overall cancer screening coverage in Peru was less than 50% and decreased during the COVID-19 pandemic period (2020–2021). The regions with more white and mestizo women had higher coverage than those with Quechua or Aymara, and Afro-Peruvian women. Also, mammogram coverage was more unequal for women 50–69 than pap smear coverage for women 50–64. Since 2017, inequality has declined, but during and after the pandemic period in Peru, it increased, especially in Quechua or Aymara, and Afro-Peruvian women (Fig. 5).

## Discussion

It was found that the coverage for cervical cancer screening with pap testing in Peruvian women exceeded the national target of 60% in 2017 and 80% in 2023 for all women except for those Afro-Peruvians [3, 14]. This could be related to the high mortality rate for cervical cancer in Peru, an expression of barriers to accessing cancer care in some ethnic groups and [2, 31]. Notably, women without higher education, with lower incomes, and residing in rural areas or outside of the capital are at higher risk [32, 33]. The findings also show that non-white and mestizo women undergo cervical and breast cancer screenings less frequently than other ethnic groups, highlighting inequalities in cancer screening utilization [34–37]. Socioeconomic inequalities faced by Quechua or Aymara women further exacerbate these inequities, suggesting systemic gaps within the Peruvian healthcare system such as limited access to healthcare facilities, particularly for these women, that belonging to native Peruvian groups, who faced challenges such as language barriers, discrimination from healthcare professionals, and differing perspectives on health prevention [18, 27, 38].

Only one third of women aged 50 to 69 receive mammography screening, contributing to the high prevalence of advanced-stage breast cancer in Peruvian women. Socioeconomic inequity exacerbates challenges in breast cancer screening, diagnosis, and treatment [25, 39], with inequalities particularly pronounced among women with lower education levels, lower income, and residing in rural areas where less than 20% of mammograms are performed. White or mestizo women show lower inequalities in all cancer screening evaluations considered in this study, compared to Quechua, Aymara, or Afro-Peruvian women. The inequality in mammography screening underscores the influence of cancer-related beliefs, stigma, and discomfort with screening among non-white or mestizo women [35, 40, 41]. Clinical breast examination emerges as an alternate strategy with higher coverage among Peruvian women, highlighting the importance of boosting awareness through self-examination initiatives [42].

Despite previous research highlighting the exacerbating effects of poverty, lower education, and a lack of health insurance on cancer screening inequalities [43–46], this study finds that Quechua, Aymara, and Afro-Peruvian women continue to reside in areas marked by significant inequalities in screening services due to these factors. These adverse sociodemographic conditions contribute to increased inequity in cancer screening coverage across Peruvian regions, particularly in those with more women identified as Quechua, Aymara, and Afro-Peruvian [18, 27, 38]. Additionally, this situation could be closer of Brazil context, where breast cancer mortality rates are increasing among Afro-American women [47]. Thus, efforts to raise awareness about cancer screening must be evaluated, with a focus on implementing measures to overcome women’s screening challenges and establishing systems to facilitate timely cancer diagnosis and treatment initiation [42, 48].

Assessing inequalities in cancer screening among Peruvian women is important, and this study reveals higher breast and cervical cancer screening inequalities among Quechua, Aymara, and Afro-Peruvian women residing in rural and non-capital areas. This could be attributed to greater access to healthcare and cancer treatment facilities, specifically in urban areas like Lima, the capital city of Peru, which exhibits the highest rates of pap smear testing, clinical breast examination, and mammography coverage [19, 20, 49]. However, the centralism in Peru and the limited availability of resources, staff, and infrastructure for cancer diagnosis in rural areas underscore the challenges of implementing more sensitive and specific screening tests for breast and cervical cancer [40, 50, 51].

Furthermore, women with higher education levels demonstrate lower ethnic inequalities in the utilization of mammography and pap smear tests, emphasizing the importance of educational interventions in enhancing cancer knowledge and raising awareness about participating in breast and cervical cancer prevention programs [52–55]. Such programs should encompass not only screening tests but also immunization against the human papillomavirus to prevent cervical cancer malignant lesions [56, 57]. Additionally, efforts to enhance vaccination coverage and reinforce the regular practice of breast self-examination among women for improve early cancer detection [58, 59].

In the period covered in this research on breast and cervical cancer screening inequalities, the COVID-19 pandemic emerged in Peru since 2020 [60, 61]. It was found that overall cancer screening coverage declined dramatically in this period (2020 and 2021), perhaps due to the confinement and limitations of cancer screening programs, affecting more Quechua, Aymara, and Afro-Peruvian women. The immunization measures for controlling COVID-19 in Peru, could also impact in a minor increase in cancer screening coverage since 2022 [62]. Quechua, Aymara, and Afro-Peruvian women had a stable trend of higher mammography and pap test coverage inequality compared to white or mestizo Peruvian women, who exhibited lower inequality in 2022. While there was no consistent pattern of ethnic inequalities in utilization of breast and cervical cancer screening services, Quechua, Aymara, and Afro-Peruvian women exhibited a tendency towards greater inequality during and after the COVID-19 pandemic compared to other ethnic groups.

The inequality measures used in this research approximate the unequal coverage of cancer screening for women belonging to different ethnic groups in the 25 Peruvian regions. However, the GINI coefficient may yield higher inequality estimations for certain sociodemographic conditions due to improper registration of Quechua, Aymara, and Afro-Peruvian groups in certain regions [63]. On the other hand, the common trend in inequalities in the use of these cancer screening assessments among Peruvian women estimated with the SII may be an expression of the unequal distribution of wealth across some specific groups that face adverse sociodemographic conditions [64]. The higher poverty in Peru after COVID-19 pandemic could explains the increase in health inequalities [65].

The study’s limitations in examining ethnic discrepancies in cancer screening coverage and utilization among Peruvian women were due to social desirability bias, where some women gave good responses without testing. Some women may not remember if these screenings were for cancer or may have had other breast and cervical cancer screening tests, such as DNA testing for human papillomavirus, visual inspections using acetic acid or Lugol’s iodine on cervix samples, or magnetic resonance and ultrasound scans for breast cancer suspicion. This study did not examine whether certain women were checked for breast or cervical cancer based on family history, past diagnosis or treatment, or screening awareness. However, the DHS’s Spanish-only questions and lack of a multicultural approach underrepresent specific ethnic groups.

## Conclusion

In conclusion, it was identified ethnic inequalities in breast and cervical cancer screening coverage for women across Peruvians regions. Moreover, those women who faced adverse sociodemographic conditions like lower education levels and living in rural or non-capital places led to significant inequalities in the utilization of mammography and pap smear tests. These inequalities were higher among Quechua, Aymara, and Afro-Peruvian women compared to those identifying as white or mestizo. This is concerning, given the increase in these inequalities during and after the COVID-19 pandemic hit the Peruvian population.

### Electronic supplementary material

Below is the link to the electronic supplementary material.

Supplementary Material 1

## Data Availability

The dataset supporting the conclusions of this article is available in the INEI repository: https://proyectos.inei.gob.pe/microdatos/
