# Supplementary material for: Ethnic inequalities in coverage and use of women’s cancer screening in Peru
Source: BMC Womens Health. 2024 Jul 24;24:418. doi: 10.1186/s12905-024-03225-6 (PMC11267911; doi:10.1186/s12905-024-03225-6)
Supplement: Supplementary file 1 — Supplementary Material 1 [file 12905_2024_3225_MOESM1_ESM.docx]

**Appendices**

**Appendix 1.-** Participant selection flowchart from the DHS in Peru since 2017 to 2023

**DHS:** Demographic and Health Survey.

**Number of participants registered in the DHS between 2017-2023
(N = 217796)**

**Participants aged between 30 and 69 years in the DHS between 2017-2023 (n = 135989)**

**Exclusion of participants under 30 years or above 69 years (n= 81807)**

**Women aged between 30 and 69 years in the DHS between 2017-2023 (n = 70677)**

**Male participants surveyed (n = 65312)**

**Women not applicable for individual survey
(n = 223)**

**Women who underwent a general cancer screening test
(n = 19839)**

**Women who have had a clinical breast examination by a health professional or physician (n = 13852)**

**Women who have had a Pap smear performed by a health care professional or physician (n = 12211)**

**Women who have had a mammogram performed by a health care professional or physician (n = 5737)**

**DHS 2017
(N = 33072)**

**DHS 2018
(N = 14462)**

**DHS 2019
(N = 33114)**

**DHS 2020
(N = 34185)**

**DHS 2021
(N = 33731)**

**DHS 2022
(N = 35080)**

**DHS 2023
(N = 34152)**

**Appendix 2.** Inequality in women's cancer screening coverage among the 25 Peruvians regions


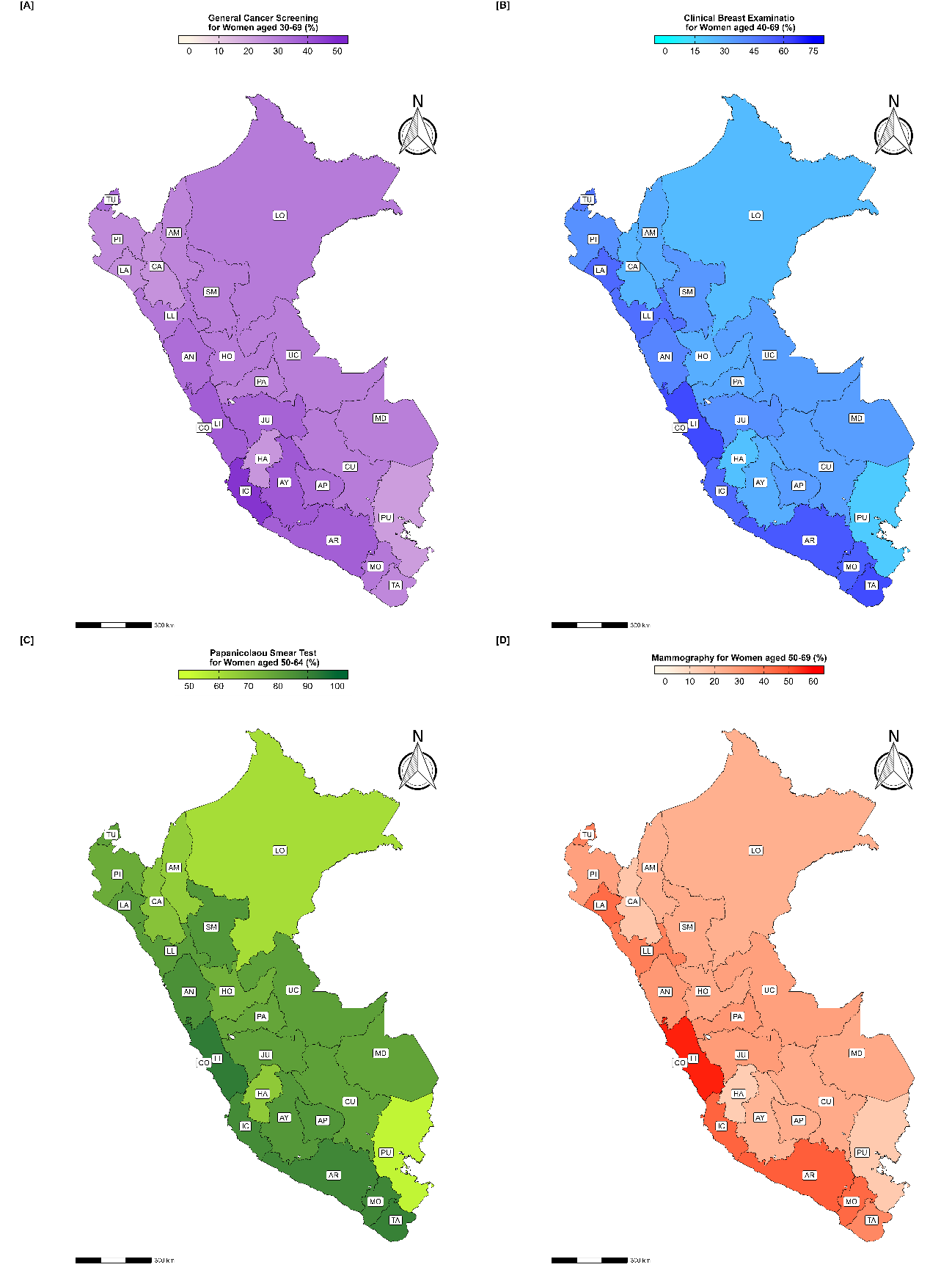


**LI:** Lima, the capital of Peru**, CO:** Callao, region near the capital.
